# Shatavari supplementation in postmenopausal women alters the skeletal muscle proteome and pathways involved in training adaptation

**DOI:** 10.1007/s00394-023-03310-w

**Published:** 2024-01-12

**Authors:** Mary F. O’Leary, Sarah R. Jackman, Joanna L. Bowtell

**Affiliations:** Department of Public Health and Sport Sciences, Faculty of Health and Life Sciences, University of Exeter, Exeter, UK

**Keywords:** *Asparagus racemosus*, Skeletal muscle, Nutrition, Proteomics

## Abstract

**Purpose:**

Shatavari is an understudied, widely available herbal supplement. It contains steroidal saponins and phytoestrogens. We previously showed that six weeks of shatavari supplementation improved handgrip strength and increased markers of myosin contractile function. Mechanistic insights into shatavari’s actions are limited. Therefore, we performed proteomics on vastus lateralis (VL) samples that remained from our original study.

**Methods:**

In a randomised double-blind trial, women (68.5 ± 6 years) ingested either placebo or shatavari (equivalent to 26,500 mg/d fresh weight) for six weeks. Tandem mass tag global proteomic analysis of *VL* samples was conducted (*N* = 7 shatavari, *N* = 5 placebo). Data were normalized to total peptides and scaled using a reference sample. Data were filtered using a 5% FDR. For each protein, the pre to post supplementation difference was expressed as log2 fold change. Welch’s *t* tests with Benjamini–Hochberg corrections were performed for each protein. Pathway enrichment (PADOG, CAMERA) was interrogated in Reactome (v85).

**Results:**

No individual protein was significantly different between supplementation conditions. Both PADOG and CAMERA indicated that pathways related to (1) Integrin/MAPK signalling, (2) metabolism/insulin secretion; (3) cell proliferation/senescence/DNA repair/cell death; (4) haemostasis/platelets/fibrin; (5) signal transduction; (6) neutrophil degranulation and (7) chemical synapse function were significantly upregulated. CAMERA indicated pathways related to translation/amino acid metabolism, viral infection, and muscle contraction were downregulated.

**Conclusion:**

Our analyses indicate that shatavari may support muscle adaptation responses to exercise. These data provide useful signposts for future investigation of shatavari’s utility in conserving and enhancing musculoskeletal function in older age.

**Trial registration:**

NCT05025917 30/08/21, retrospectively registered.

**Supplementary Information:**

The online version contains supplementary material available at 10.1007/s00394-023-03310-w.

## Introduction

Shatavari (*Asparagus racemosus* Willd.) is a herb that has been widely used in Ayurveda, but without much empirical evidence for its effectiveness. Recently, evidence has emerged that shatavari supplementation may increase skeletal muscle strength. We showed that six weeks of shatavari supplementation significantly improved handgrip strength in postmenopausal women [1]. Further, myosin regulatory light chain phosphorylation, which is a known marker of improved myosin contractile function, was increased in the vastus lateralis following shatavari supplementation. Shatavari also increased the phosphorylation of Akt^ser473^, but not other traditional molecular markers of muscle protein synthesis e.g., phosphorylation of p70S6k^Thr389^, 4EBP1^Thr37/46^ or S6^Ser240/244^.

An earlier study showed that shatavari promoted strength gains in young men during eight weeks of bench press training [2]. However, this study did not attempt to elucidate possible mechanisms of shatavari action. There is, therefore, a need for greater mechanistic insight into the actions of shatavari in skeletal muscle. This will provide molecular corroboration of the observed functional effects, guide further research questions and ensure that these questions are targeted at the most appropriate athletic and patient populations.

Estradiol receptor activation by phytoestrogens could improve muscle strength, particularly in the estradiol deficient postmenopausal population that we studied previously. Indeed, this was the rationale for our original work [1]. Estradiol is known to improve myosin binding function and muscle force production [3]. Hormone replacement therapy improves muscle strength in postmenopausal women [4]. However, the work of Anders et al. in young men suggests that the mechanism of shatavari action in skeletal muscle is likely more complicated than estradiol-mimicking effects alone [2].

The bioactive constituents of shatavari are manifold. They comprise steroidal saponins (shatavarins I–IV), known antioxidants such as racemosides, racemosol, racemofuran and asparagamine A and phytoestrogens e.g., rutin, kaempferol, genistein, daidzein and quercetin [5]. Identifying and quantifying the bioactive constituents of shatavari that are responsible for its functional effects remains a distinct challenge. However, the complex bioactive constituents of shatavari may evoke skeletal muscle adaptations that are relevant to endurance performance and metabolic health. For example, kaempferol can increase mitochondrial complex IV activity and adenosine triphosphate (ATP) production in hypoxic C2C12 myotubes [6]. Rutin supplementation of rats with diet-induced obesity increases mitochondrial size, mitochondrial DNA content and the expression of genes implicated in mitochondrial biogenesis [7]. However, the evidence surrounding the effects of individual compounds in these scenarios is disparate. Such evidence also remains centred around animal and in vitro work that is of variable quality, and it is unclear whether such effects would translate to specific human scenarios. Bioactive supplements and compounds that are known to have anti-inflammatory and anti-oxidant effects are understudied in relation to long term human muscle function and adaptations to resistance training. The dearth of evidence in this area may stem from observations that high doses of some dietary supplements e.g. anti-oxidant vitamins (e.g. vitamin C and vitamin E) adversely affect resistance training adaptations, although the balance of evidence is equivocal [8–10]. We have recently shown that a polyphenol-rich tart cherry supplement is unlikely to act as a direct anti-oxidant, rather 8 days of supplementation upregulates endogenous skeletal muscle glutathione peroxidase expression [11]. This suggests that the effects of complex bioactive supplements might differ from effects that have been observed when high doses of exogenous antioxidant vitamins have been administered. Resistance training in young men increases the skeletal muscle expression of antioxidant gene terms [12]. Further, resistance training in older men increases skeletal muscle gene expression of superoxide dismutase 1 (SOD1) and 2 (SOD2), catalase (CAT) and glutathione peroxidases (GPXs), although protein levels were unchanged and enzyme activities varied. The enzymatic activity of CAT increased, whereas GPX activity decreased [13]. This limited evidence suggests that complex bioactive supplements may support or at the very least not impair, resistance training adaptations. In the case of shatavari, this remains speculative.

Shatavari may represent a novel, effective, low cost and safe nutraceutical intervention for the prevention and treatment of age-related muscle weakness. Such weakness is present in all older adults within our ageing population, with the risk of overt sarcopenia increasing with age [14]. This age-related decline in muscle mass and function and the associated morbidity and mortality may cost the UK healthcare sector £2.5 billion per year [15]. Research into shatavari’s effects on skeletal muscle function remains in its infancy and questions of efficacy and mechanisms of action must be resolved.

Therefore, to provide mechanistic insights and signposts for future work, we performed tandem mass tagged global proteomics on skeletal muscle samples that remained from our original shatavari supplementation study in postmenopausal women. Given our previous work and the work of Anders et al. [2] we hypothesised that shatavari supplementation might increase the levels of proteins associated with muscle protein synthesis and myosin function.

## Materials and methods

### Study design and supplementation protocol

The skeletal muscle samples that were used for these analyses were obtained as part of a previously published study [1]. This double-blind, placebo-controlled parallel design study was approved by the University of Exeter’s Sport and Health Sciences Research Ethics Committee (190206/B/01) and an amendment to this approval was secured to allow for the analyses presented in this paper. All participants had consented to the use of their samples in future work.

Briefly, participants attended a screening and consent visit during which eligibility was again confirmed and informed consent was obtained. Participants returned for visit two having fasted overnight. They completed tests of leg strength. These consisted of a warm-up, three sets of three maximal isokinetic repetitions (concentric and eccentric effort) and three maximum voluntary contractions with the knee joint fixed at 90 degrees of flexion. All sets were separated by one minute of rest.

A vastus lateralis skeletal muscle biopsy was performed [1]. A researcher who was not involved in any other aspect of the study randomised participants to receive either placebo or shatavari supplementation for six weeks using a random number generator. Participants consumed two capsules per day containing either placebo (*N* = 10; magnesium stearate, 1000 mg total) or shatavari (*N* = 10; 1000 mg powder equivalent to 26,500 mg fresh weight shatavari root). The capsules were visually identical and did not have an odour.

Compliance was assessed by providing an excess of capsules and counting the number that remained at the end of the study period. No participant missed more than three doses over the six-week supplementation period. Visit three took place at the conclusion of this supplementation period and was identical to visit two.

### Proteomic analysis

We powered our analyses in the original (not the current) study to be able to detect a 1.6-fold change in myogenin protein expression with 10 participants per group. We did this using standard deviations derived from unpublished internal immunoblotting data from older adults. For this follow-up work, a complete set of skeletal muscle samples were available for analysis from *N* = 7 shatavari supplemented individuals and *N* = 5 placebo supplemented individuals. Skeletal muscle (c. 15 mg) was placed in microcentrifuge tubes with 250 μL of radioimmunoprecipitation assay (RIPA) buffer (Pierce 89,900 RIPA buffer, ThermoFisher Scientific) containing protease and phosphatase inhibitors (Pierce A32961 Protease and Phosphatase Inhibitor EDTA-free mini tablet, ThermoFisher Scientific). Samples were homogenised for 1 min using a bead homogeniser (Speedmill Plus, Analytik Jena AG). Samples were removed to clean microcentrifuge tubes and vortexed thoroughly before incubation on ice for 30 min, with occasional vortexing. Samples were centrifuged for 10 min at 8000 g at 4 °C. The supernatant was removed to a clean microcentrifuge tube and the pellet was discarded. Protein concentrations were determined by bicinchoninic acid (BCA) assay (Pierce 23,225 BCA Protein Assay Kit, ThermoFisher Scientific) according to the manufacturer’s instructions.

### TMT labelling, high pH reversed-phase chromatography

Aliquots of 50 µg of each sample were digested with trypsin (1.25 µg trypsin; 37 °C, overnight), labelled with Tandem Mass Tag (TMTpro) sixteen plex reagents according to the manufacturer’s protocol (Thermo Fisher Scientific, Loughborough, LE11 5RG, UK) and the labelled samples pooled.

An aliquot of 200ug of the pooled sample was desalted using a SepPak cartridge according to the manufacturer’s instructions (Waters, Milford, Massachusetts, USA). Eluate from the SepPak cartridge was evaporated to dryness and resuspended in buffer A (20 mM ammonium hydroxide, pH 10) prior to fractionation by high pH reversed-phase chromatography using an Ultimate 3000 liquid chromatography system (Thermo Fisher Scientific). In brief, the sample was loaded onto an XBridge BEH C18 Column (130 Å, 3.5 µm, 2.1 mm × 150 mm, Waters, UK) in buffer A and peptides eluted with an increasing gradient of buffer B (20 mM Ammonium Hydroxide in acetonitrile, pH 10) from 0 to 95% over 60 min. The resulting fractions (20 in total) were evaporated to dryness and resuspended in 1% formic acid prior to analysis by nano-LC MSMS using an Orbitrap Fusion Lumos mass spectrometer (Thermo Scientific).

### Nano-LC mass spectrometry

High pH RP fractions were further fractionated using an Ultimate 3000 nano-LC system in line with an Orbitrap Fusion Lumos mass spectrometer (Thermo Scientific). In brief, peptides in 1% (vol/vol) formic acid were injected onto an Acclaim PepMap C18 nano-trap column (Thermo Scientific). After washing with 0.5% (vol/vol) acetonitrile 0.1% (vol/vol) formic acid peptides were resolved on a 250 mm × 75 μm Acclaim PepMap C18 reverse phase analytical column (Thermo Scientific) over a 150 min organic gradient, using 7 gradient segments (1–6% solvent B over 1 min., 6–15% B over 58 min., 15–32%B over 58 min., 32–40%B over 5 min., 40–90%B over 1 min., held at 90%B for 6 min and then reduced to 1%B over 1 min.) with a flow rate of 300 nl min − 1. Solvent A was 0.1% formic acid and Solvent B was aqueous 80% acetonitrile in 0.1% formic acid. Peptides were ionized by nano-electrospray ionization at 2.0 kV using a stainless-steel emitter with an internal diameter of 30 μm (Thermo Scientific) and a capillary temperature of 300 °C.

All spectra were acquired using an Orbitrap Fusion Lumos mass spectrometer controlled by Xcalibur 3.0 software (Thermo Scientific) and operated in data-dependent acquisition mode using an SPS-MS3 workflow. FTMS1 spectra were collected at a resolution of 120,000, with an automatic gain control (AGC) target of 200,000 and a max injection time of 50 ms. Precursors were filtered with an intensity threshold of 5000, according to charge state (to include charge states 2–7) and with monoisotopic peak determination set to Peptide. Previously interrogated precursors were excluded using a dynamic window (60 s ± 10 ppm). The MS2 precursors were isolated with a quadrupole isolation window of 0.7 m/z. ITMS2 spectra were collected with an AGC target of 10,000, max injection time of 70 ms and CID collision energy of 35%.

For FTMS3 analysis, the Orbitrap was operated at 50,000 resolution with an AGC target of 50,000 and a max injection time of 105 ms. Precursors were fragmented by high energy collision dissociation (HCD) at a normalised collision energy of 60% to ensure maximal TMT reporter ion yield. Synchronous Precursor Selection (SPS) was enabled to include up to 10 MS2 fragment ions in the FTMS3 scan.

### Data analysis

The raw data files were processed and quantified using Proteome Discoverer software v2.4 (Thermo Scientific) and searched against the UniProt Human database (downloaded January 2022: 178,486 entries) using the SEQUEST HT algorithm. Peptide precursor mass tolerance was set at 10 ppm, and MS/MS tolerance was set at 0.6 Da. Search criteria included oxidation of methionine (+ 15.995 Da), acetylation of the protein N-terminus (+ 42.011 Da) and Methionine loss plus acetylation of the protein N-terminus (− 89.03 Da) as variable modifications and carbamidomethylation of cysteine (+ 57.0214) and the addition of the TMTpro mass tag (+ 304.207) to peptide N-termini and lysine as fixed modifications. Searches were performed with full tryptic digestion and a maximum of 2 missed cleavages were allowed. The reverse database search option was enabled and all data were filtered to satisfy false discovery rate (FDR) of 5%.

### Bioinformatics

Data were filtered to include only proteins for which 10 of 12 possible data points were available. These filtered data were used in all subsequent analyses. For each protein, the pre to post supplementation difference was calculated for each participant as a fold change. These values were log2 transformed and were used in all subsequent comparisons between groups. A Welch’s *t*-test (*t*.test(group1, group2, var.equal = FALSE)), with a Benjamini–Hochberg correction (p.adjust(p_values, method = "BH")) was then performed to compare treatment groups (R version 4.2.3). Further analyses were performed using Reactome v85.

Decisions made in the design of an enrichment analysis can have a profound effect on the results [16]. We performed differential gene expression analysis using Reactome’s ‘Pathway Analysis with Down-weighting of Overlapping Genes’ (PADOG) and ‘Correlation Adjusted MEan RAnk gene set test’ (CAMERA) algorithms. PADOG uses log2 transformed quantitative proteomics data and calculates a gene set score and down-weights genes/proteins that appear in many molecular pathways. As inter-protein correlations can skew analyses, CAMERA estimates the inter-protein correlation from the data, and uses it to adjust the protein set test statistic. CAMERA and PADOG have been suggested to have complementary strengths and weaknesses, therefore, both methods were used to indicate which findings are most likely to be robust [16]. We report false discovery rates (FDR) and fold change (FC) values for Reactome analyses. These are calculated by the Reactome algorithm as part of the CAMERA and PADOG analyses. Using details given in the Reactome Pathway Database, significantly altered (FDR < 0.05) pathways from both analyses were manually classified into biological themes: Transmission Across Chemical Synapses, Metabolism/Insulin Secretion, Neutrophil Degranulation, Haemostasis/Platelets/Fibrin, Cellular Signal Transduction, Integrin/MAPK/BRAF/RAF Signalling, Cell proliferation/senescence/DNA repair/cell death. Detail regarding which sub-pathways were manually collapsed into these biological themes is available in the Online Resource 1.

To visualise the network, the 500 most prominently up and downregulated proteins (as determined by the magnitude of log2 fold change between the pre and post supplementation biopsies) were visualised in Cytoscape v3.10.0 using stringApp v2.0.1 [17]. The full STRING network was used with a high confidence threshold of 0.7. Log2 fold change values were used to style the network, indicating expression of individual proteins in the shatavari condition relative to the placebo control. Network clustering was performed using the Markov clustering (MCL) implementation in the clusterMaker2 Cytoscape app; the inflation value was set to 4.0.

## Results

### Proteome changes

Four thousand, four hundred and eleven proteins were detected at quantifiable levels, with 3121 of these proteins being detected in all samples (3132 in at least 10/12 samples). No protein was differentially expressed between placebo and shatavari supplementation conditions when a Benjamini–Hochberg correction was applied to the results of Welch’s *t* tests.

Differential expression analysis found that pathways related to (1) Integrin/MAPK signalling, (2) metabolism/insulin secretion; (3) cell proliferation/senescence/DNA repair/cell death; (4) haemostasis/platelets/fibrin; (5) signal transduction; (6) neutrophil degranulation and (7) chemical synapse function were significantly upregulated (Table 1). The full list of pathways, along with their FDR and FC values are presented in Online Resources 2, 3 and 4.

**Table 1:** Upregulated biological themes

	PADOG/CAMERA agreement	Additional PADOG only pathways	Additional CAMERA only pathways	Mean FDR (PADOG)	Mean logFC (PADOG)	Number of pathways
Integrin/MAPK/BRAF/RAF signalling	Yes	Yes	No	0.0125	0.47	13
Metabolism/insulin secretion	Yes	Yes	No	0.0212	0.30	5
Cellproliferation/senescence/DNA repair/cell death	No	Yes	No	0.0286	0.58	5
Haemostasis/platelets/fibrin	Yes	Yes	Yes	0.0303	0.46	2
Cellular signal transduction, general	Yes	Yes	Yes	0.0310	0.52	4
Neutrophil degranulation	Yes	Yes	No	0.0375	0.46	3
Transmission across chemical synapses	Yes	Yes	Yes	0.0405	0.54	4

Pathways related to these themes were found to be significantly upregulated following Reactome's PADOG and/or CAMERA analysis. 'PADOG/CAMERA agreement' indicates that the same pathway/s related to this theme were found to be upregulated (FDR < 0.05) via both analysis methods. This table also indicates where additional pathways related to the theme were found to be upregulated by PADOG/CAMERA only

Only CAMERA (not PADOG) analysis indicated that any pathways were downregulated. Notably, pathways related to translation/amino acid metabolism, viral infection, and muscle contraction contained a large number of downregulated proteins (Table 2). The full list of pathways, along with their FDR and fold change values are presented in Online Resource 2.

**Table 2:** Downregulated biological themes

Pathway cluster	Number of pathways	Mean FDR	Mean logFC
Translation/amino acid metabolism	22	0.0000098	− 0.25
Viral infection	4	0.0087824	− 0.19
Muscle contraction	2	0.0001548	− 0.23
ROBO signalling/axonal guidance	1	0.0000003	− 0.14
DNA damage/telomeres/stress induced senescence	1	0.0087824	− 0.19

Pathways related to these themes were found to be significantly downregulated (FDR < 0.05) following Reactome's CAMERA analysis

**Fig. 1:**
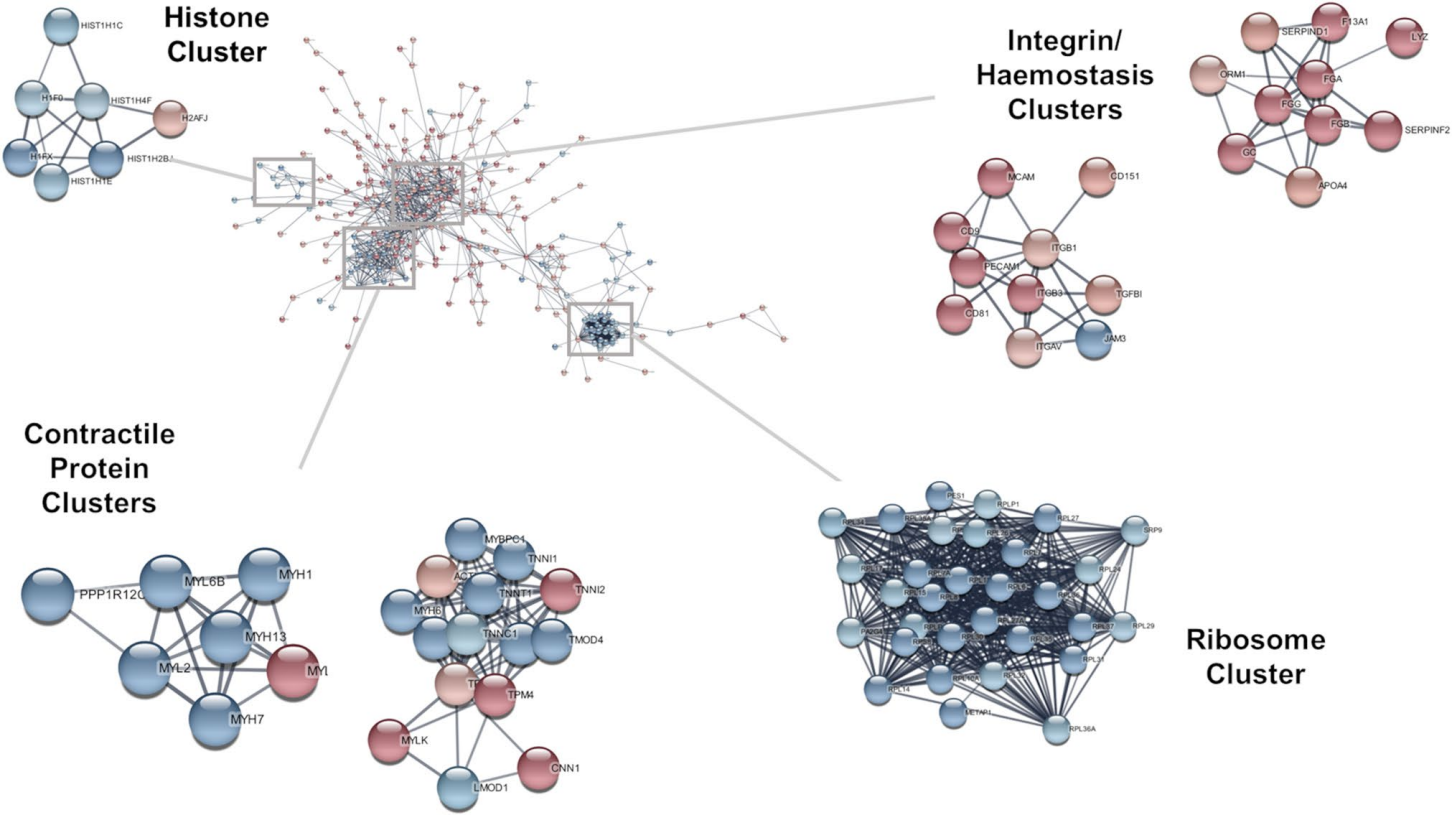
STRING network of proteins of the 500 most prominently up and downregulated proteins in skeletal muscle following six weeks shatavari supplementation. Log2 fold changes (of pre to post supplementation mean differences) between placebo and shatavari supplemented groups for each protein were mapped to the nodes using a blue−white−red gradient. Proteins with fewer than two interaction partners within the network are omitted. To aid readability, protein names are indicated by their official gene symbol

Visualisation of the 500 most prominently up and downregulated proteins and Markov clustering highlighted ‘ribosome’, ‘contractile protein’ and ‘histone’ clusters containing proteins downregulated by shatavari supplementation. It also highlighted ‘integrin’ and ‘haemostasis’ clusters which contained proteins upregulated by shatavari supplementation. Clusters containing at least 7 nodes are displayed in Fig. 1.

## Discussion

**Fig. 2:**
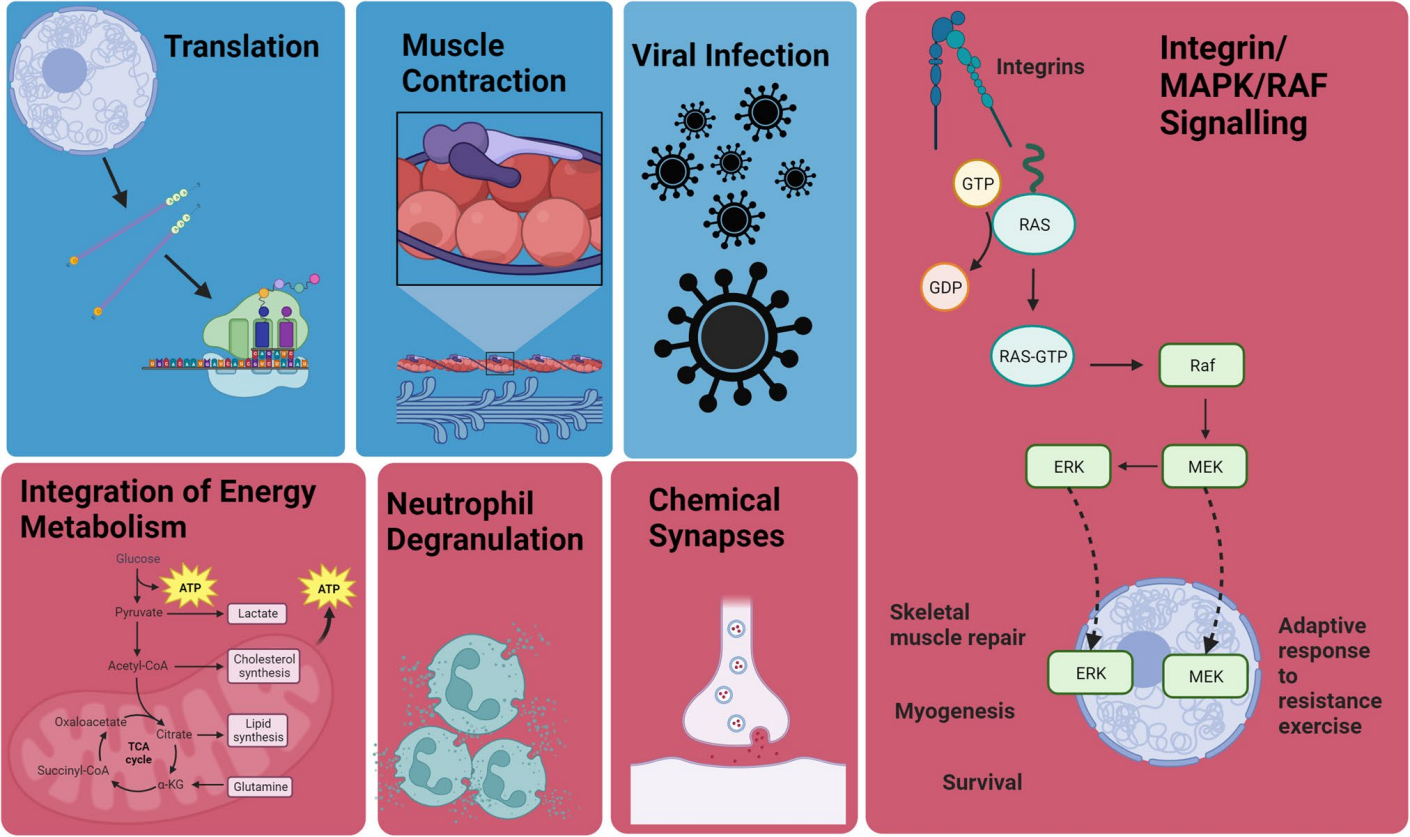
Some of the prominenent up/downregulated biological themes identified by in skeletal muscle following six weeks shatavari supplementation. Red = upregulated compared to placebo, blue = downregulated compared to placebo

Here, we performed tandem mass tagged global proteomics on skeletal muscle samples that remained from a previous 6 week shatavari supplementation study in postmenopausal women. Our previous study showed that six weeks of shatavari supplementation improved handgrip strength and increased the phosphorylation of the myosin regulatory light chain, a molecular indicator of improved myosin function. Further, the work of Anders et al. demonstrated that shatavari supplementation during a resistance training programme improved bench press strength, repetitions to failure at 70% one-repetition maximum and a more rapid accumulation of training load [2]. Therefore, we hypothesised that shatavari supplementation might increase the levels of proteins associated with muscle protein synthesis and myosin function. We demonstrated particularly prominent changes (shatavari vs placebo) in pathways involved in integrin/MAPK signalling, energy metabolism, neutrophil degranulation, chemical synapse transmission (all increased); muscle contraction, protein translation, and viral infection (all decreased) (Fig. 2).

We note that the skeletal muscle biopsies used for these analyses were taken approximately 30 min after a moderate bout of resistance exercise, which comprised a warm-up, nine (three sets of three) maximum isokinetic contractions and three maximum isometric voluntary contractions. The results can, therefore, most accurately be considered representative of the muscle proteome shortly after a bout of acute resistance exercise. This is critical in considering the implications of our analyses for training adaptations. However, it is transcriptional responses, epigenetic changes and post translational modifications of proteins, rather than translational changes that are traditionally thought to predominate in this timeframe [18]. Therefore, the extent to which the bout of resistance exercise altered the ‘resting’ proteome is unknown and warrant separate investigation in a future study.

It is notable that we demonstrated a shatavari-induced upregulation of pathways involved in integrin and MAPK signalling. Integrins act as force sensors at the sarcolemma—the muscle cell membrane—connecting the extracellular matrix to the skeletal muscle contractile apparatus. Integrins are implicated in skeletal muscle hypertrophic responses to exercise and generation of external forces [19]. The mechanism/s that underpin this remain opaque, although it appears that this occurs independently of mammalian target of rapamycin complex 1 (mTORC1) [20]. Evidence is emerging that an integrin–integrin-linked kinase–RICTOR–Akt (Rapamycin-insensitive companion of mTOR = RICTOR; protein kinase B = Akt) pathway may mediate muscle adaptations to exercise [20, 21]. The results of our original targeted immunoblotting analyses of these samples found a significant increase in Akt phosphorylation, with no changes in the phosphorylation status of effectors of muscle protein synthesis downstream of mTORC1 (Ribosomal protein S6, Ribosomal protein S6 kinase beta-1, Eukaryotic translation initiation factor 4E-binding protein 1). As these biopsies were taken shortly after a moderate bout of resistance exercise, this suggests that shatavari supplementation may indeed enhance signalling in pathways other than mTORC1 that are responsive to resistance exercise. Shatavari also enhanced the expression of proteins in pathways involved in mitogen-activated protein kinases (MAPK) signalling. The MAPK pathway is responsible for transmitting diverse extracellular signals to allow for a co-ordinated cellular response. Extracellular signal-regulated kinase 1/2 (ERK), c-Jun N-terminal kinase (JNK) and p38 are the ‘big three’ effector kinases in human skeletal muscle. ERK and JNK have long been known to have central roles and complex roles in co-ordinating skeletal muscle repair and myogenesis [22–24]. Recent work has identified a key role for JNK in the adaptive response to resistance exercise, via Mothers against decapentaplegic homolog 2 (SMAD2) inhibition of myostatin [25]. ERK is activated by endurance [26] and resistance [27] exercise. We note the enrichment of proteins that are mapped to pathways involving Ras and Raf (Fig. 2). Upstream of ERK 1/2, the GTPase Ras activates the c-Raf protein kinases which activates Mitogen-activated protein kinase kinases 1 and 2 (MEK1/2) and in turn, ERK 1/2 [28]. Pathways related to Haemostasis/Platelets/Fibrin were found to be enriched (Table 1). Notably, eight of 11 proteins detected in our samples which are associated with the Reactome pathways ‘integrin signalling’ and ‘MAP2K and MAPK activation’ are also considered by Reactome in assessing changes in the ‘MAP2K and MAPK activation’ and ‘Hemostasis’ pathways. These proteins are MAPK2, fibrinogen beta chain,talin-1, integrin beta-3, ras-related protein Rap-1A, fibrinogen alpha chain, ras-related protein Rap-1b and von Willebrand factor. It is unclear whether the shatavari-induced changes that we observed in the expression of these proteins are more important within skeletal muscle with regard to integrin/MAPK signal transduction or haemostasis/clot formation. Any changes in haemostasis/clot formation following shatavari consumption warrant investigation; this may represent an important ‘off target’ effect.

CAMERA analysis indicated that expression of proteins in pathways related to protein translation, metabolism of RNA and metabolism of proteins was downregulated. It is possible that shatavari supplementation may induce a state of reduced protein turnover within skeletal muscle. Skeletal muscle mass may be gained or lost through an imbalance between muscle protein synthesis (MPS) and breakdown (MPB). In the context of age-related loss of skeletal muscle mass (sarcopenia), a blunted MPS response to nutritional and exercise stimuli is thought to be one major aetiological factor. Further, nutritional strategies to counteract this age-related imbalance have traditionally been found to succeed via ‘correcting’ this blunted MPS [29]. However, this does not preclude a positive net protein balance being achieved via a state of reduced muscle protein turnover. Counter to this hypothesis is a lack of changes observed in pathways related to MPB.

It is also possible that the difference in the expression of ribosomal proteins between shatavari and placebo supplemented groups could be explained as a homeostatic response to a shatavari-induced improvement in ribosome translational efficiency. In this scenario, the amount of protein synthesised per unit of mRNA is increased by shatavari, resulting in a compensatory reduction in the amount of translational machinery (capacity) within the skeletal muscle. The roles and time-course of translational efficiency and capacity on resistance-training adaptations remain speculative [30]. Modern analytical approaches, such as the use of deuterium oxide (D_2_O) to quantify RNA synthesis MPS and MPB may allow these issues to be resolved [31, 32]. Caution is required in considering these findings as the PADOG and CAMERA approaches were not in agreement. However, it is notable that STRING MCL analysis identified a prominent cluster of ribosomal proteins with reduced expression in the shatavari condition compared to the placebo condition.

Interestingly, the data suggest an upregulation of skeletal muscle processes related to cell proliferation/senescence/DNA repair/cell death (Table 1, Online Resource 2). This suggests that any alteration in protein turnover is not necessarily accompanied by a lack of skeletal muscle maintenance or ‘quality control’. We note that PADOG analysis suggested an increase in proteins associated with the pentose phosphate pathway, specifically regulation of this pathway by an important regulator of cellular anti-oxidant responses [33], the transcription factor nuclear factor erythroid 2-related factor 2 (Nrf2). The pentose phosphate pathway is a branch of the main glycolytic pathway, producing NADPH and ribose 5-phosphate which are central to nucleic acid, fatty acid nucleotide and non-essential amino acid synthesis. NADPH is also important in facilitating the conversion of glutathione to its reduced form; this is a critical component of cellular antioxidant defence [34]. The functional and adaptive value of these observations remains to be explored.

Perhaps counter-intuitively, our results identified that levels of proteins in pathways related to muscle contraction were reduced. In our original study of these participants, we described an increase in handgrip strength in the shatavari-supplemented group. This was accompanied by an increase in molecular indicators of improved muscle contractility (myosin regulatory light chain phosphorylation). In this proteomic study, the expression of the kinase responsible for this phosphorylation was (non-significantly) increased, (myosin light-chain kinase expression, Fig. 1). Although this increase in expression was not significant in a proteomic study with low numbers of participants, it is notable that the expression most other muscle proteins that were networked with myosin light-chain kinase expression following MCL clustering were decreased by the shatavari condition compared to placebo. These proteins included a subunit of myosin phosphatase, myosin light chain, myosin heavy chain, actin and troponin isoforms. It is possible that this represents a homeostatic downregulation of these proteins/pathways in response to a short-term improvement in muscle contractility.

Proteins associated with chemical synapse transmission pathways were observed to be increased following shatavari supplementation (Table 1). The human neuromuscular junction remains structurally stable across the lifespan, despite animal models suggesting the contrary [35]. However, the link between anatomical and functional stability is unclear. Intramuscular electromyogram (EMG) data suggest that synaptic transmission becomes compromised in older age, although this may be somewhat modifiable by exercise [36–39]. A prominent feature of neuromuscular ageing is denervation of muscle fibres and the growth of new axons to re-innervate and rescue these fibres. This is known as axonal sprouting. This process is imperfect and leads to larger motor units, with reduced discharge rates, increased recruitment thresholds and impaired muscle performance. There is some evidence to suggest that older athletes do display enhanced axonal sprouting and re-innervation of muscle fibres [40, 41]. Our data suggest that shatavari may have the potential to improve neuromuscular function in older populations and support such adaptations to resistance or endurance training. This is speculative and requires further study.

We note with interest the increase in proteins associated with metabolic pathways, including several which are implicated in carbohydrate metabolism, insulin secretion and the interconversion of nucleotide di- and triphosphates. The implications of these observations for skeletal muscle ATP availability, carbohydrate metabolism and whole-body glycaemic control under resting and exercise conditions are unclear. However, the value of these data in providing signposts for future work that might not have otherwise been considered is evident.

Our results suggest that shatavari may possess immunomodulatory properties; indeed, limited evidence had suggested this prior to the COVID-19 pandemic. Shatavari administration to mice who were immunised against diphtheria, tetanus and pertussis resulted in a c. 16% greater antibody titre response at 14 days post-inoculation [42]. Administration of shatavari extract to mice with paclitaxel (PTX)-induced myelosuppression normalised leukocyte and neutrophil counts [43]. In recent years, several in silico studies have suggested that shatavari has the potential to inhibit COVID-19 via the hemagglutinin-acetylesterase (HE) glycoprotein receptor which is required for host infection [44–46]. This recent in silico work has yet to be confirmed in reliable in vitro or in vivo work. However, the immunomodulatory properties of shatavari should be explored further.

We did not analyze the bioactive constituents of our shatavari root supplement. High quality quantitative characterisations of such supplements are lacking, with many investigations and reviews providing qualitative, rather than quantitative information [5, 47]. One recent high-quality study exists and notably, they identified 33 different steroidal saponins [48]. These saponins were particularly enriched within shatavari root compared to the leaf and the fruit. They also identified 16 triterpene saponins that had previously not been described in shatavari. Additionally, 31 flavonoids were described. The discovery of new compounds that had previously not been described in the literature demonstrates the importance of further detailed characterisations of shatavari root. Characterisations of whole food dietary supplements are typically incomplete and provide only a snapshot of prominent, known compounds that may not necessarily be responsible for the biological effects of the supplement. For this reason, we believe that it is important to first do work to prove/refute efficacy for such supplements that are already being sold over the counter and used by consumers. This is what we have done in our recent work. Identification of the individual compound/s within shatavari root that exert biological effects then becomes a worthy research question requiring complex analytical work. This should now be a priority in the shatavari supplementation research field. Indeed, it remains possible that the non-phytoestrogenic components of shatavari may contribute to its effects within skeletal muscle; Anders et al. [2] showed that shatavari is effective in improving bench press strength in young men. Therefore, we also anticipate that future work will focus on delineating which compounds within shatavari are important in improving muscle strength and whether there are sex-specific differences in this regard. This will be important for the potential application of our findings to older men who are at risk of sarcopenia.

These analyses were conducted using samples that remained from previously published work. The original study was designed to detect 1.6-fold changes in the expression of proteins analysed by immunoblotting, a technique that is well-recognised as having quantitative limitations [49, 50]. Insufficient *VL* sample remained for proteomics analysis for several participants. Therefore, in this follow-up study, the participant numbers are smaller. However, pathway analyses improve statistical power by aggregating changes in all proteins involved in a pathway/process [51]. Indeed, these sample numbers have proved sufficient to identify pathway-level proteomic differences between the supplementation groups. Perhaps unsurprisingly—given the need to correct rigorously for multiple comparisons—these low sample numbers were not sufficient to detect changes in individual proteins between conditions. We acknowledge that a risk of false and non-generalisable conclusions does exist, although the ‘dual’ controls of both a placebo condition and pre-post supplementation changes in each group minimise this. Critically in this regard, the proteomic differences between these supplementation groups are consistent with the limited published evidence of the effects of shatavari on skeletal muscle function. Specifically, increased muscle strength is accompanied by downregulation of proteins in pathways related to metabolism of proteins, amino acids and RNA and upregulation of proteins in pathways related to integrin/MAPK signalling, cell growth, metabolism, apoptosis, and chemical synapse transmission (Fig. 2). Therefore, we consider these data to be a novel, valuable and timely contribution to this emerging area of bioactives research. Most importantly, these data will influence the design and direction of future shatavari investigations. Given our findings in postmenopausal women, we suggest that the effect of shatavari on muscle function could be investigated in sarcopenic populations, both in the presence and absence of resistance training. The combination of shatavari with protein supplementation may also have merit, whether the effects of these dietary interventions can be additive in improving muscle strength and hypertrophy.

### Supplementary Information

Below is the link to the electronic supplementary material.

Supplementary file1 (XLSX 13 KB)

Supplementary file2 (DOCX 27 KB)

Supplementary file3 (XLSX 268 KB)

Supplementary file4 (XLSX 232 KB)

## Data Availability

The datasets used and/or analyzed during the current study are available from Open Research Exeter: 10.24378/exe.4926.
