# Supplementary material for: Shatavari supplementation in postmenopausal women alters the skeletal muscle proteome and pathways involved in training adaptation
Source: Eur J Nutr. 2024 Jan 12;63(3):869–79. doi: 10.1007/s00394-023-03310-w (PMC10948523; doi:10.1007/s00394-023-03310-w)
Supplement: Supplementary file 2 — Supplementary file2 (DOCX 27 KB) [file 394_2023_3310_MOESM2_ESM.docx]

**Shatavari Supplementation in Postmenopausal Women Alters the Skeletal Muscle Proteome and Pathways Involved in Training Adaptation**

Mary F O’Leary^1*^, Sarah R Jackman^1^, Joanna L Bowtell^1^

^1^ Faculty of Health and Life Sciences, Department of Public Health and Sport Sciences, University of Exeter, Exeter, United Kingdom.

*Correspondence: m.oleary@exeter.ac.uk; Tel: 0044 1392 72 475

ORCID:

MFOL 0000-0003-1845-9568

SRJ 0000-0003-2928-8266

JLB 0000-0002-4281-9212

**Key Words:** asparagus racemosus, skeletal muscle, nutrition, proteomics

| **Online Resource Table 2.1.** Differential expression analysis of the effects of six weeks shatavari supplementation on protein expression in human skeletal muscle performed using Reactome’s PADOG algorithm. Pathways presented are those with a false discovery rate (FDR) of < 0.05 | | | |
| --- | --- | --- | --- |
| **Pathway Name** | **Direction  of Change** | **FDR** | **Fold Change** |
| Release of apoptotic factors from the mitochondria | Up | 0.013 | 0.739 |
| Activation, translocation and oligomerization of BAX | Up | 0.013 | 0.739 |
| NTRK3 as a dependence receptor | Up | 0.013 | 0.739 |
| OAS antiviral response | Up | 0.040 | 0.665 |
| p130Cas linkage to MAPK signaling for integrins | Up | 0.009 | 0.656 |
| Adenylate cyclase inhibitory pathway | Up | 0.049 | 0.593 |
| Integrin signaling | Up | 0.010 | 0.561 |
| GRB2:SOS provides linkage to MAPK signaling for Integrins | Up | 0.010 | 0.561 |
| GABA receptor activation | Up | 0.029 | 0.540 |
| TP53 Regulates Transcription of Genes Involved in Cytochrome C Release | Up | 0.040 | 0.536 |
| RHOB GTPase cycle | Up | 0.019 | 0.531 |
| ADP signalling through P2Y purinoceptor 12 | Up | 0.042 | 0.516 |
| Signal amplification | Up | 0.042 | 0.516 |
| GABA B receptor activation | Up | 0.042 | 0.516 |
| Activation of GABAB receptors | Up | 0.042 | 0.516 |
| Formyl peptide receptors bind formyl peptides and many other ligands | Up | 0.030 | 0.497 |
| MAP2K and MAPK activation | Up | 0.011 | 0.492 |
| Signaling by high-kinase activity BRAF mutants | Up | 0.011 | 0.492 |
| Signaling by RAF1 mutants | Up | 0.011 | 0.492 |
| Platelet Aggregation (Plug Formation) | Up | 0.015 | 0.485 |
| TP53 Regulates Transcription of Genes Involved in G2 Cell Cycle Arrest | Up | 0.034 | 0.482 |
| G alpha (z) signalling events | Up | 0.039 | 0.469 |
| Adrenaline,noradrenaline inhibits insulin secretion | Up | 0.032 | 0.457 |
| Signaling by moderate kinase activity BRAF mutants | Up | 0.013 | 0.456 |
| Signaling by RAS mutants | Up | 0.013 | 0.456 |
| Paradoxical activation of RAF signaling by kinase inactive BRAF | Up | 0.013 | 0.456 |
| Signaling downstream of RAS mutants | Up | 0.013 | 0.456 |
| Signaling by BRAF and RAF1 fusions | Up | 0.008 | 0.437 |
| Thrombin signalling through proteinase activated receptors (PARs) | Up | 0.046 | 0.437 |
| G alpha (s) signalling events | Up | 0.044 | 0.435 |
| GPER1 signaling | Up | 0.044 | 0.435 |
| ADORA2B mediated anti-inflammatory cytokines production | Up | 0.044 | 0.435 |
| Insulin effects increased synthesis of Xylulose-5-Phosphate | Up | 0.028 | 0.434 |
| TP53 Regulates Transcription of Cell Death Genes | Up | 0.043 | 0.413 |
| RHOC GTPase cycle | Up | 0.035 | 0.410 |
| Regulation of insulin secretion | Up | 0.042 | 0.392 |
| G-protein mediated events | Up | 0.048 | 0.391 |
| NFE2L2 regulates pentose phosphate pathway genes | Up | 0.023 | 0.385 |
| Oncogenic MAPK signaling | Up | 0.016 | 0.371 |
| PI and PC transport between ER and Golgi membranes | Up | 0.049 | 0.363 |
| Interleukin-4 and Interleukin-13 signaling | Up | 0.023 | 0.333 |
| Post-translational modification: synthesis of GPI-anchored proteins | Up | 0.046 | 0.325 |
| Integration of energy metabolism | Up | 0.009 | 0.304 |
| Integrin cell surface interactions | Up | 0.025 | 0.235 |
| Iron uptake and transport | Up | 0.047 | 0.228 |
| Neutrophil degranulation | Up | 0.042 | 0.204 |
| Cellular response to heat stress | Up | 0.027 | 0.119 |
| Disease | Up | 0.025 | 0.079 |
| Interconversion of nucleotide di- and triphosphates | Up | 0.005 | 0.003 |

| **Online Resource Table 2.2.** Differential expression analysis of the effects of six weeks shatavari supplementation on protein expression in human skeletal muscle performed using Reactome’s CAMERA algorithm. Pathways presented are those with a false discovery rate (FDR) of < 0.05. | | | |
| --- | --- | --- | --- |
| **Pathway Name** | **Direction  of Change** | **FDR** | **Fold Change** |
| **Down** | | | |
| Response of EIF2AK4 (GCN2) to  amino acid deficiency | Down | 5.4441E-15 | -0.315 |
| Selenocysteine synthesis | Down | 7.824E-15 | -0.314 |
| Major pathway of rRNA processing  in the nucleolus and cytosol | Down | 7.824E-15 | -0.314 |
| Nonsense-Mediated Decay (NMD) | Down | 1.8211E-14 | -0.281 |
| Nonsense Mediated Decay (NMD)  enhanced by the Exon Junction Complex | Down | 1.8211E-14 | -0.281 |
| Peptide chain elongation | Down | 1.8211E-14 | -0.301 |
| Nonsense Mediated Decay (NMD)  independent of the Exon Junction Complex | Down | 1.8211E-14 | -0.302 |
| SRP-dependent cotranslational protein targeting to membrane | Down | 1.8985E-14 | -0.270 |
| rRNA processing | Down | 1.8985E-14 | -0.284 |
| rRNA processing in the nucleus and cytosol | Down | 1.8985E-14 | -0.295 |
| Formation of a pool of free 40S subunits | Down | 3.4782E-14 | -0.274 |
| Eukaryotic Translation Termination | Down | 3.4782E-14 | -0.292 |
| Viral mRNA Translation | Down | 3.6464E-14 | -0.297 |
| L13a-mediated translational silencing of Ceruloplasmin expression | Down | 5.1584E-14 | -0.260 |
| Eukaryotic Translation Elongation | Down | 7.8291E-14 | -0.276 |
| GTP hydrolysis and joining of the 60S ribosomal subunit | Down | 1.4702E-13 | -0.252 |
| Cellular response to starvation | Down | 2.5047E-13 | -0.262 |
| Influenza Viral RNA Transcription and Replication | Down | 4.4414E-13 | -0.260 |
| Eukaryotic Translation Initiation | Down | 5.7751E-12 | -0.224 |
| Cap-dependent Translation Initiation | Down | 5.7751E-12 | -0.224 |
| Selenoamino acid metabolism | Down | 9.5689E-11 | -0.227 |
| Influenza Infection | Down | 4.2374E-10 | -0.207 |
| Regulation of expression of SLITs and ROBOs | Down | 2.4852E-09 | -0.159 |
| Signaling by ROBO receptors | Down | 5.9097E-07 | -0.118 |
| Translation | Down | 1.6554E-06 | -0.077 |
| Metabolism of amino acids and derivatives | Down | 5.8591E-05 | -0.066 |
| Metabolism of RNA | Down | 0.00015482 | -0.048 |
| Striated Muscle Contraction | Down | 0.00015482 | -0.226 |
| DNA Damage/Telomere Stress Induced Senescence | Down | 0.01057368 | -0.266 |
| Viral Infection Pathways | Down | 0.01577344 | -0.013 |
| **Up** | | | |
| p130Cas linkage to MAPK signaling for integrins | Up | 4.051E-05 | 0.656 |
| MAP2K and MAPK activation | Up | 0.0003 | 0.492 |
| Signaling by high-kinase activity BRAF mutants | Up | 0.0003 | 0.492 |
| Signaling by RAF1 mutants | Up | 0.0003 | 0.492 |
| Signaling by BRAF and RAF1 fusions | Up | 0.0004 | 0.437 |
| Integrin signaling | Up | 0.0010 | 0.561 |
| GRB2:SOS provides linkage to MAPK signaling for Integrins | Up | 0.0010 | 0.561 |
| Signaling by moderate kinase activity BRAF mutants | Up | 0.0010 | 0.456 |
| Signaling by RAS mutants | Up | 0.0010 | 0.456 |
| Paradoxical activation of RAF signaling by kinase inactive BRAF | Up | 0.0010 | 0.456 |
| Signaling downstream of RAS mutants | Up | 0.0010 | 0.456 |
| Platelet activation, signaling and aggregation | Up | 0.0024 | 0.293 |
| Platelet Aggregation (Plug Formation) | Up | 0.0035 | 0.485 |
| Oncogenic MAPK signaling | Up | 0.0039 | 0.371 |
| Common Pathway of Fibrin Clot Formation | Up | 0.0096 | 0.642 |
| Formation of Fibrin Clot (Clotting Cascade) | Up | 0.0096 | 0.529 |
| Hemostasis | Up | 0.0106 | 0.240 |
| Platelet degranulation | Up | 0.0111 | 0.306 |
| Response to elevated platelet cytosolic Ca2+ | Up | 0.0148 | 0.296 |
| GABA receptor activation | Up | 0.0202 | 0.540 |
| Transmission across Chemical Synapses | Up | 0.0321 | 0.299 |
| Signal Transduction | Up | 0.0347 | 0.187 |
| Integration of energy metabolism | Up | 0.0380 | 0.304 |
| Neuronal System | Up | 0.0380 | 0.267 |
| RAC1 GTPase cycle | Up | 0.0408 | 0.336 |
| RHOB GTPase cycle | Up | 0.0420 | 0.531 |
| Regulation of insulin secretion | Up | 0.0457 | 0.392 |
| Neutrophil degranulation | Up | 0.0474 | 0.204 |
